# First Integrals of Shear-Free Fluids and Complexity

**DOI:** 10.3390/e23111539

**Published:** 2021-11-19

**Authors:** Sfundo C. Gumede, Keshlan S. Govinder, Sunil D. Maharaj

**Affiliations:** 1 Astrophysics Research Centre, School of Mathematics, Statistics and Computer Science, University of KwaZulu-Natal, Private Bag X54001, Durban 4000, South Africa; gumedeSC@mut.ac.za (S.C.G.); govinder@ukzn.ac.za (K.S.G.); 2 Department of Mathematical Sciences, Mangosuthu University of Technology, P.O. Box 12363, Jacobs 4026, South Africa

**Keywords:** shear-free fluids, Einstein field equations, first integrals

## Abstract

A single master equation governs the behaviour of shear-free neutral perfect fluid distributions arising in gravity theories. In this paper, we study the integrability of $y_{xx}=f\left(x\right)y^{2},$ find new solutions, and generate a new first integral. The first integral is subject to an integrability condition which is an integral equation which restricts the function f(x). We find that the integrability condition can be written as a third order differential equation whose solution can be expressed in terms of elementary functions and elliptic integrals. The solution of the integrability condition is generally given parametrically. A particular form of $f\left(x\right)∼\frac{1}{x^{5}}{\left(1-\frac{1}{x}\right)}^{-15/7}$ which corresponds to repeated roots of a cubic equation is given explicitly, which is a new result. Our investigation demonstrates that complexity of a self-gravitating shear-free fluid is related to the existence of a first integral, and this may be extendable to general matter distributions.

## 1. Introduction

In many studies, the concept of complexity has been applied to topics such as entropy and information. An intriguing approach is to also utilize this concept in self-gravitating systems. Herrera [1] suggested that complexity in gravity would be studied by the definition of a minimal complexity factor. This approach may also be applied to dissipative fluids in general relativity with applications to compact stars, neutron stars, and radiating objects in the strong gravity regime. Several investigations have been initiated involving the concept of complexity in self-gravitating systems in general relativity and some modified theories of gravity [2,3,4,5,6,7,8,9,10,11,12]. Jasim et al. [13] studied a strange star model in a special case of Lovelock theory, namely Einstein–Gauss–Bonnet gravity, and showed that such theories are consistent with the concept of complexity. General matter distributions including dissipative effects are necessary to analyse relativistic self-gravitating fluids. Shear-free matter distributions arise as a special case and deserve special attention because of their applicability to stellar models, and they have been used to model both static and radiating stars. Therefore, in this investigation, we consider the behaviour of shear-free fluids in a spherical spacetime. Our results indicate that it is possible to find new first integrals which provide insight into the behaviour of the self-gravitating fluids. Our approach may help in generating a general relationship between first integrals, extended to a general shearing relativistic matter distribution, and the complexity of a self-gravitating relativistic fluid.

Seeking exact solutions to the Einstein field equations has been the subject of study in many astrophysical and cosmological applications. Such solutions may be used to model inhomogeneous processes in systems of galaxies and the broader universe [14]. Exact solutions to the field equations have also been used to model and investigate properties of observable phenomena such as relativistic stars [15] as well as expanding and contracting spherical stars [16]. Dissipative fluids in general relativity are of particular importance because of various applications in astrophysics and the description of radiating stars. The general framework for the study of physically acceptable dissipating systems in spherical symmetry was undertaken in several works [17,18,19,20,21]. Some particular exact models have been found using this framework [22,23,24,25]. The special case of vanishing shear provides new insights into the behaviour of gravity, and some particular radiating stellar models have been generated [26,27,28,29]. Our approach in this paper is to find a general result, namely a first integral, in a shear-free fluid without having to specify the gravitational potentials.

When seeking exact solutions to the Einstein field equations, it is usual to assume spherical symmetry for spacetimes and the absence of shear for the matter distribution. These assumptions greatly simplify the field equations while ensuring that the results are still physically meaningful. Spherically symmetric shear-free solutions have been used to model many physical applications. Some of the classes of these solutions were obtained by Stephani [30], Srivastava [31], Sussman [32,33], and Maharaj et al. [34]. Brassel et al. [35] found gravitational potentials for shear-free heat conducting fluids in terms of elementary functions in a recent treatment. However, it is important to note that most of the known exact solutions to the Einstein field equations are not shearing [36]. This is largely due to the fact that the shear-free condition introduces an additional equation which needs to be solved. The Einstein field equations for spherically symmetric shear-free neutral matter comprise a system of nonlinear partial differential equations. We will show how this system of equations can be further reduced to the single Emden–Fowler equation $y_{xx}=f\left(x\right)y^{2}$. The first general solution to this Emden–Fowler equation in general relativity was found by Kustaanheimo and Qvist [37] for a specified form of the function f(x). Other classes of solutions were found later by Stephani [30] and Srivastava [31]. Sussman [32], Wafo Soh and Mahomed [38], and Maharaj et al. [39] found further classes of solutions by assuming that the spacetime is invariant under a conformal Killing vector. Another recent treatment of this problem was given by Maharaj et al. [34].

The shear-free condition is often applied in the study of radiating stars, gravitational collapse, and relativistic astrophysical processes. The vanishing shear assumption leads to a simplification of the field equations. Note that the homogeneous expansion rate and shear-free condition are the classical analogue of homologous fluids in the Newtonian limit. This means that the shear-free assumption in general relativity is well justified. However, it is important to point out that the shear-free fluids may be unstable due to perturbations arising from anisotropy and dissipation. Herrera et al. [40] investigated the conditions when an initial shear-free configuration continues to be shear-free as the system evolves. Pressure anisotropy and dissipation affect the propagation of the relativistic fluid. These quantities play a role in the realistic modelling involving gravitational collapse and should be contained in a stable model.

In this paper, we analyse the integrability of and find exact solutions to the Emden–Fowler equation using an ad hoc method that was previously shown to be useful [34]. In Section 2, we show how the field equations for the spherically symmetric nonstatic shear-free metric reduce to the equation $y_{xx}=f\left(x\right)y^{2}$. We obtain its first integral in Section 3. This first integral is subject to the integrability condition which we study in Section 4. In Section 5, we find the functional form of the function f(x) and give the corresponding first integral.

## 2. Shear-Free Fluids

The metric for a shear-free, perfect fluid in the comoving and isotropic coordinate system $\left(x^{a}\right)=\left(t,r,\theta ,\phi \right)$ is given by

(1) $$ds^{2}=-e^{2\nu (t,r)}dt^{2}+e^{2\lambda (t,r)}\left[dr^{2}+r^{2}\left(d\theta^{2}+\sin^{2}\theta \phi^{2}\right)\right],$$

where $e^{2\nu }$ and $e^{2\lambda }$ are the gravitational potentials. The Einstein field equations take the form

(2a) $$\begin{array}{ccc} \mu & = & 3\frac{{\lambda_{t}}^{2}}{e^{2\nu }}-\frac{1}{e^{2\lambda }}\left(2\lambda_{rr}+\lambda_{r}^{2}+\frac{4\lambda_{r}}{r}\right), \end{array}$$

(2b) $$\begin{array}{ccc} p & = & \frac{1}{e^{2\nu }}\left(-2\lambda_{tt}-3\lambda_{t}^{2}+2\nu_{t}\lambda_{t}\right)+\frac{1}{e^{2\lambda }}\left(\lambda_{r}^{2}+2\nu_{r}\lambda_{r}+\frac{2\nu_{r}}{r}+\frac{2\lambda_{r}}{r}\right), \end{array}$$

(2c) $$\begin{array}{ccc} p & = & \frac{1}{e^{2\nu }}\left(-2\lambda_{tt}-3\lambda_{t}^{2}+2\nu_{t}\lambda_{t}\right)+\frac{1}{e^{2\lambda }}\left(\nu_{rr}+\nu_{r}^{2}+\frac{\nu_{r}}{r}+\frac{\lambda_{rr}}{r}+\lambda_{rr}\right), \end{array}$$

(2d) $$\begin{array}{ccc} 0 & = & \nu_{r}\lambda_{t}-\lambda_{tr}. \end{array}$$

The quantities μ and *p* represent the energy density and pressure, respectively. The subscripts *r* and *t* in Equation (2) above represent partial derivatives with respect to *r* and t, respectively.

The condition of pressure isotropy is obtained by equating (2b) and (2c). The resulting equation can be easily integrated once with respect to time which results in an arbitrary function of integration, g(r). We can also integrate (2d) once with respect to *r* and obtain another arbitrary function of integration, h(t). Using these simplifications, we can now write the system (2) in the form

(3a) $$\begin{array}{ccc} \mu & = & 3e^{2h}-e^{-2\lambda }\left(2\lambda_{rr}+\lambda_{r}^{2}+\frac{4\lambda_{r}}{r}\right), \end{array}$$

(3b) $$\begin{array}{ccc} p & = & \frac{1}{\lambda_{t}}{\left[e^{-2\lambda }\left(\lambda_{r}^{2}+\frac{2\lambda_{r}}{r}\right)-e^{2h}\right]}_{t}, \end{array}$$

(3c) $$\begin{array}{ccc} e^{\nu } & = & \lambda_{t}e^{-h}, \end{array}$$

(3d) $$\begin{array}{ccc} e^{\lambda }\left(\lambda_{rr}-\lambda_{r}^{2}-\frac{\lambda_{r}}{r}\right) & = & -g(r). \end{array}$$

The functions *h* and *g* need to be specified in order to find exact solutions for the field equations. Thereafter, the metric function λ can be obtained by solving (3d), while the remaining metric function ν then follows directly from (3c). Equation (3a,b) then define the energy density μ and the isotropic pressure *p*, respectively. It is clear that the pivotal equation is (3d).

Using the transformation

$$\begin{array}{ccc} x & = & r^{2},\\ y(x,t) & = & e^{-\lambda },\\ f(x) & = & \frac{g}{4r^{2}}, \end{array}$$

Equation (3d) reduces to

(4) $$y_{xx}=f\left(x\right)y^{2},$$

as first shown by Kustaanheimo and Qvist [37]. Equation (4) is the master equation governing the gravitational dynamics of a shear-free fluid in general relativity.

There have been a number of studies seeking solutions of field Equation (4). However, the solution is known for only a few forms of f(x). The solution with

$$f\left(x\right)={\left(a+bx+cx^{2}\right)}^{-5/2},$$

was given by Kustaanheimo and Qvist [37]. Solutions with

$$f\left(x\right)=x^{-20/7},x^{-15/7},e^{x},$$

were found by Stephani [30]. General analyses of Equation (4) were completed by Wafo Soh and Mahomed [41], Maharaj et al. [34], and Stephani et al. [36]. A charged generalization was studied by Kweyama et al. [42].

Our approach mirrors Maharaj et al. [34], who extended an idea of Srivastava [31]. We briefly outline that approach here. We can integrate the left hand side of (4) once directly and the right hand side by repeated applications of ‘integration-by-parts’. This eventually yields the first integral [34]

(5) $$\psi_{0}\left(t\right)=-y_{x}+f_{I}y^{2}-2f_{II}yy_{x}+2f_{III}y_{x}^{2}+2\left[{\left(ff_{II}\right)}_{I}-\frac{1}{3}K_{0}\right]y^{3},$$

where $\psi_{0}\left(t\right)$ is an arbitrary function of integration, $K_{0}$ is an arbitrary constant,

(6) $$f_{I}=\int fdx,$$

and we have the integrability condition

(7) $$2ff_{III}+3{\left(ff_{II}\right)}_{I}=K_{0}.$$

This equation was then thoroughly analysed by Maharaj et al. [34] to find new exact solutions including the explicit forms of f(x) given by

(8) $$f\left(x\right)=\frac{48}{343}{\left(-\frac{7b}{2}\right)}^{6/7}{\left(x-x_{0}\right)}^{15/7},$$

which led to (5). Note that *b* and $x_{0}$ are arbitrary constants of integration.

## 3. A New First Integral

We now apply the approach of Maharaj et al. [34] but with one important difference. We observe that when (4) is multiplied by *x*, it becomes

(9) $$xy_{xx}=xfy^{2}.$$

If we now define

$$\bar{f}=xf,$$

then we can write (9) in the form

(10) $$xy_{xx}=\bar{f}y^{2}.$$

We can still integrate the left hand side explicitly once while the right hand side of (10) is simply the right hand side of (4) with *f* relabelled as $\bar{f}$. Thus, the Maharaj et al. [34] integration will apply to the right hand side of (10) as well. We obtain

(11) $$xy_{x}-y={\bar{f}}_{I}y^{2}-2\int {\bar{f}}_{I}yy_{x}dx-\phi_{1}\left(t\right),$$

where for convenience we have used

(12) $${\bar{f}}_{I}=\int \bar{f}dx,$$

and $\phi_{1}\left(t\right)$ is an arbitrary function of integration. Note the subtle difference between (6) and (12).

Integrating ${\bar{f}}_{I}yy_{x}$ by parts and using (4), we obtain

(13) $$xy_{x}-y={\bar{f}}_{I}y^{2}-2{\bar{f}}_{II}yy_{x}+2\int {\bar{f}}_{II}y_{x}^{2}dx+2\int f{\bar{f}}_{II}y^{3}dx-\phi_{1}\left(t\right).$$

Integrating $f{\bar{f}}_{II}y^{3}$ and ${\bar{f}}_{II}y_{x}^{2},$ by parts again, we obtain

(14) $$\begin{array}{ccc} xy_{x}-y & = & {\bar{f}}_{I}y^{2}-2{\bar{f}}_{II}yy_{x}+2{\bar{f}}_{III}y_{x}^{2}+2{\left(f{\bar{f}}_{II}\right)}_{I}y^{3}\\ & & -2\left[\int \left[2f{\bar{f}}_{III}+3{\left(f{\bar{f}}_{II}\right)}_{I}\right]y^{2}y_{x}dx\right]-\phi_{1}\left(t\right). \end{array}$$

The integral on the right hand side of (14) can be evaluated if

(15) $$2f{\bar{f}}_{III}+3{\left(f{\bar{f}}_{II}\right)}_{I}=K_{1},$$

where $K_{1}$ is a constant. This equation can be written as a differential equation which still needs to be solved (see later).

We now have the expression

(16) $$\begin{array}{ccc} \phi_{1}\left(t\right) & = & y-xy_{x}+{\bar{f}}_{I}y^{2}-2{\bar{f}}_{II}yy_{x}+2{\bar{f}}_{III}y_{x}^{2}+2\left[{\left(f{\bar{f}}_{II}\right)}_{I}-\frac{1}{3}K_{1}\right]y^{3}, \end{array}$$

where $\phi_{1}\left(t\right)$ is an arbitrary function of integration. We then observe that (16) is another new first integral of (4), provided that condition (15) is satisfied. (It is important to emphasize that (15) is different from (7) since ${\bar{f}}_{I}=\int xf\left(x\right)dx$).

## 4. Integrability Conditions

To complete the analysis, we need to determine the form of the function f(x) (or $\bar{f}\left(x\right)$). In an attempt to seek the form of the function *f*, the integral Equation (15) can be transformed into an ordinary differential equation. It is easier to solve the differential equation rather than the integral equation. Differentiating (15), we obtain

(17) $$2f_{x}{\bar{f}}_{III}+5f{\bar{f}}_{II}=0,$$

which can be written as

(18) $$2\left[{\bar{f}}_{x}-\frac{1}{x}\bar{f}\right]{\bar{f}}_{III}+5\bar{f}{\bar{f}}_{II}=0,$$

which contains $\bar{f}$ only. Now, setting

$$\bar{L}\equiv {\bar{f}}_{III},$$

we eliminate $\bar{f}$ in (18) to give the differential equation

(19) $$2\left[{\bar{L}}_{xxxx}-\frac{1}{x}{\bar{L}}_{xxx}\right]\bar{L}+5{\bar{L}}_{x}{\bar{L}}_{xxx}=0.$$

We can integrate (19) once to obtain

(20) $${\bar{L}}_{xxx}=C_{0}x{\bar{L}}^{-5/2},$$

where $C_{0}$ is a constant of integration.

We observe that the third-order ordinary differential Equation (20) is equivalent to the integrability condition (15). Integrating (20) repeatedly, we find $\bar{L}$, and hence $\bar{f}\left(x\right)$. Solving the nonlinear differential Equation (20), we obtain

(21) $$\begin{array}{ccc} x^{2}{\bar{L}}^{-1} & = & {\bar{C}}_{3}+{\bar{C}}_{2}\left(\int x{\bar{L}}^{-\frac{3}{2}}dx\right)+{\bar{C}}_{1}{\left(\int x{\bar{L}}^{-\frac{3}{2}}dx\right)}^{2}+{\bar{C}}_{0}{\left(\int x{\bar{L}}^{-\frac{3}{2}}dx\right)}^{3}, \end{array}$$

where ${\bar{C}}_{0}=-\frac{C_{0}}{3}$ and the remaining constants are arbitrary. Therefore, the third-order Equation (20) has been completely integrated (see Appendix A for details on the integration process).

In general, we can write the solution parametrically. For convenience, we let

$$u=\int x{\bar{L}}^{-\frac{3}{2}}dx,$$

so that (21) becomes

$$x^{2}u_{x}={\left({\bar{C}}_{3}+{\bar{C}}_{2}u+{\bar{C}}_{1}u^{2}+{\bar{C}}_{0}u^{3}\right)}^{\frac{3}{2}}.$$

In the above equation, the variables separate, and we can write

(22) $$x_{0}-\frac{1}{x}=\int \frac{du}{{\left({\bar{C}}_{3}+{\bar{C}}_{2}u+{\bar{C}}_{1}u^{2}+{\bar{C}}_{0}u^{3}\right)}^{\frac{3}{2}}},$$

where $x_{0}$ is constant. Now, the function $\bar{f}\left(x\right)$ must be found satisfying the integrability condition (15). In order to find $\bar{f}\left(x\right)$ satisfying this integrability condition, it is convenient to express the solution in the parametric form

$$\begin{array}{ccc} \bar{f}\left(x\right) & = & {\bar{L}}_{xxx},\\ u_{x} & = & x{\bar{L}}^{-\frac{3}{2}},\\ x_{0}-\frac{1}{x} & = & p(u), \end{array}$$

where

$$p\left(u\right)=\int \frac{du}{{\left({\bar{C}}_{3}+{\bar{C}}_{2}u+{\bar{C}}_{1}u^{2}+{\bar{C}}_{0}u^{3}\right)}^{\frac{3}{2}}}.$$

The evaluation of the integral is determined by the values of the constants ${\bar{C}}_{0},{\bar{C}}_{1},{\bar{C}}_{2}$, and ${\bar{C}}_{3}.$

In summary, we have found a new first integral of (4) given by (16) where *f* in (4) is obtained via *L* after evaluating the integral in p(u).

## 5. Particular Solutions

The evaluation of the integral in (22) has five cases depending on the nature of the factors of the polynomial ${\bar{C}}_{3}+{\bar{C}}_{2}u+{\bar{C}}_{1}u^{2}+{\bar{C}}_{0}u^{3}.$ (Since the coefficients are arbitrary, the discriminant does not help us to reduce the options.) The five cases are

**Case** **I** —One order-three factor;
**Case** **II** —One order-one factor and one order-two factor;
**Case** **III** —Three order-one (non-repeated) factors;
**Case** **IV** —One linear factor and one quadratic factor;
**Case** **V** —No factors.

In order to illustrate the process, we provide the details of the calculation for some of these cases.

### 5.1. Case I: One Order-Three Factor

This is the simplest case as the factors are repeated. If ${\bar{C}}_{3}+{\bar{C}}_{2}u+{\bar{C}}_{1}u^{2}+{\bar{C}}_{0}u^{3}$ has one factor repeated three times, then we can write

$${\bar{C}}_{3}+{\bar{C}}_{2}u+{\bar{C}}_{1}u^{2}+{\bar{C}}_{0}u^{3}={\left(A+Bu\right)}^{3},$$

with B≠0. In this case, the integral in (22) can be evaluated to give

$$x_{0}-\frac{1}{x}=-\frac{2}{7B}{\left(A+Bu\right)}^{-7/2},$$

so that

$$\bar{L}=x^{2/3}{u_{x}}^{-2/3}=x^{2}{\left(-\frac{2}{7B}\right)}^{-6/7}{\left(x_{0}-\frac{1}{x}\right)}^{6/7}.$$

Differentiating $\bar{L}$ three times, we obtain

(23) $$f\left(x\right)=\frac{48}{343x^{5}}{\left(-\frac{2}{7B}\right)}^{-6/7}{\left(x_{0}-\frac{1}{x}\right)}^{-15/7}.$$

After reparametrisation, f(x) can be written as

(24) $$f\left(x\right)∼\frac{1}{x^{5}}{\left(1-\frac{1}{x}\right)}^{-15/7}.$$

Note that in this case the function f(x) can be found explicitly. This functional form is different from (8) in the approach of Maharaj et al. [34]. Hence, the first integral (16) for this case is a new solution to the Emden–Fowler equation. For applications, this form of the solution is probably easier to utilize in modelling as f(x) has a simple explicit form. Now, we can write the first integral (16) in terms of *x* as follows:

(25) $$\begin{array}{ccc} \phi_{1}\left(t\right) & = & y-xy_{x}+2{\left(-\frac{2}{7B}\right)}^{-6/7}{\left(x_{0}-\frac{1}{x}\right)}^{6/7}y^{2}+\frac{12}{7x}{\left(-\frac{2}{7B}\right)}^{-6/7}{\left(x_{0}-\frac{1}{x}\right)}^{-1/7}y^{2}\\ & & -\frac{6}{49x^{2}}{\left(-\frac{2}{7B}\right)}^{-6/7}{\left(x_{0}-\frac{1}{x}\right)}^{-8/7}y^{2}-4x{\left(-\frac{2}{7B}\right)}^{-6/7}{\left(x_{0}-\frac{1}{x}\right)}^{6/7}yy_{x}\\ & & -\frac{12}{7}{\left(-\frac{2}{7B}\right)}^{-6/7}{\left(x_{0}-\frac{1}{x}\right)}^{-1/7}yy_{x}+2x^{2}{\left(-\frac{2}{7B}\right)}^{-6/7}{\left(x_{0}-\frac{1}{x}\right)}^{6/7}y_{x}^{2}\\ & & +{\left[\frac{192}{343x^{4}}{\left(-\frac{2}{7B}\right)}^{-12/7}{\left(x_{0}-\frac{1}{x}\right)}^{-9/7}\right]}_{I}y^{3}+{\left[\frac{576}{2401}{\left(-\frac{2}{7B}\right)}^{-12/7}{\left(x_{0}-\frac{1}{x}\right)}^{-16/7}\right]}_{I}y^{3}\\ & & -\frac{2}{3}K_{1}y^{3}. \end{array}$$

The subscripts *I* in the Equation (25) denote a remaining integration which we have omitted for brevity. It can be observed that this first integral is different from the first integral obtained by Maharaj et al. [34].

In addition to the analysis performed by Maharaj et al. [34], we substitute the function given in (23) into the integrability condition (15) in order to find restriction(s) on the constant $K_{1}.$ This substitution implies that $K_{1}=0$ in the first integral (25). Similarly, in the Maharaj et al. [34] solution, substituting (8) into the integrability condition (7) yields $K_{0}=0$ in the first integral (5).

### 5.2. Case II: One Order-One Factor and One Order-Two Factor

If ${\bar{C}}_{3}+{\bar{C}}_{2}u+{\bar{C}}_{1}u^{2}+{\bar{C}}_{0}u^{3}$ has one factor repeated, then we can write

$${\bar{C}}_{3}+{\bar{C}}_{2}u+{\bar{C}}_{1}u^{2}+{\bar{C}}_{0}u^{3}=\left(A+Bu\right){\left(u+C\right)}^{2},$$

with B≠0. In this case, the integral in (22) can be evaluated using [43] to obtain

(26) $$\begin{array}{ccc} p(u) & = & \left(\frac{15B^{2}}{4{\left(A-BC\right)}^{3}}+\frac{5B}{4u{\left(A-BC\right)}^{2}}-\frac{1}{2u^{2}\left(A-BC\right)}\right)\frac{1}{\sqrt{A+Bu-BC}}\\ & & +\frac{15B^{2}}{8{\left(A-BC\right)}^{3}}\int \frac{du}{u\sqrt{A+Bu-BC}}, \end{array}$$

where the integral can be expressed in terms of elementary functions depending on the sign of A−BC. For this case, it is not possible to obtain the function u(x) explicitly, as it is not possible to evaluate the integral on the right hand side of (26). Therefore the solution can only be given parametrically. However, the integral on the right hand side of (26) can be evaluated in special cases: for example, A=0 and C=0. If A=0, we can write

$${\bar{C}}_{3}+{\bar{C}}_{2}u+{\bar{C}}_{1}u^{2}+{\bar{C}}_{0}u^{3}=Bu{\left(u+C\right)}^{2}.$$

Using the computer package Maple [44] to evaluate the integral in (22), we obtain

$$\begin{array}{ccc} & & p\left(u\right)=-\frac{7}{4}\frac{\sqrt{Bu}}{BC^{3}\left(BC+Bu\right)}-\frac{15}{4}\frac{\arctan\left(\frac{\sqrt{Bu}}{\sqrt{BC}}\right)}{BC^{3}\sqrt{BC}}-\frac{1}{2}\frac{\sqrt{Bu}}{c^{2}{\left(BC+Bu\right)}^{2}}-\frac{2}{BC^{3}\sqrt{Bu}}. \end{array}$$

If C=0, then we have that

$${\bar{C}}_{3}+{\bar{C}}_{2}u+{\bar{C}}_{1}u^{2}+{\bar{C}}_{0}u^{3}=\left(A+Bu\right)u^{2}.$$

We evaluate the integral in (22) using Mathematica [45] to obtain

$$\begin{array}{c} p\left(u\right)=\frac{15B^{2}u^{2}+5ABu-2A^{2}}{4A^{3}u^{2}\sqrt{A+Bu}}-\frac{15B^{2}\tanh\left(\frac{\sqrt{A+Bu}}{\sqrt{A}}\right)}{4A^{7/2}}. \end{array}$$

We observe that even for these special cases of *A* and C, it is not easy to perform the inversion in order to obtain the function u(x) explicitly.

### 5.3. Case III: Three Order-One (Non-Repeated) Factors

If ${\bar{C}}_{3}+{\bar{C}}_{2}u+{\bar{C}}_{1}u^{2}+{\bar{C}}_{0}u^{3}$ has three non-repeated factors, then we can write

$${\bar{C}}_{3}+{\bar{C}}_{2}u+{\bar{C}}_{1}u^{2}+{\bar{C}}_{0}u^{3}=D\left(A-u\right)\left(B-u\right)\left(C-u\right),$$

with D≠0. In this case, with the aid of Mathematica [45], the integral in (22) can be written in terms of elliptic integrals to obtain

$$\begin{array}{ccc} p(u) & = & \frac{2[C(A-C)+B(A-B)-u(2A-C-B)]}{D^{3/2}\left(A-B\right)\left(A-C\right){\left(B-C\right)}^{2}\sqrt{\left(A-u)(B-u)(C-u\right)}}\\ & & +\frac{2[(B-C)(A+B-2C)F(\alpha ,\beta )]}{D^{3/2}{\left(A-B\right)}^{2}{\left(B-C\right)}^{2}\sqrt{{\left(A-C\right)}^{3}}}-\frac{2[\left(A^{2}+B^{2}+C^{2}-AB-AB-BC\right)E\left(\alpha ,\beta \right)]}{D^{3/2}{\left(A-B\right)}^{2}{\left(B-C\right)}^{2}\sqrt{{\left(A-C\right)}^{3}}}, \end{array}$$

where we have set

$$\alpha =\arcsin\sqrt{\frac{A-C}{A-u}},$$

and

$$\beta =\sqrt{\frac{A-B}{A-C}}.$$

In this form of the solution, the quantities F(α,β) and E(α,β) are elliptic integrals of the first and second kind, respectively. In this case of non-repeated factors, we also cannot obtain u(x) explicitly, and hence the solution can only be given in parametric form.

In cases IV and V, the integral (22) can also be evaluated using elliptic functions. However, the subsequent expressions are lengthy, and since we cannot obtain f(x) explicitly, we omit those results here.

## 6. Discussion

The Emden–Fowler equation $y_{xx}=f\left(x\right)y^{2}$ governs the behaviour of spherically symmetric shear-free uncharged fluid distributions. In this paper, we investigated the integrability of this equation and found a new class of exact solutions. This equation has several applications in general relativity and other areas of mathematical physics. We multiplied the Emden–Fowler equation by an integrating factor *x* and used integration by parts to obtain the first integral which is given by (16), subject to the integrability condition (15). The first integral and the integrability condition are different from the corresponding ones given in [34]. We were able to solve the integral Equation (15) by first transforming it to a third-order ordinary differential Equation (20) whose solution was given by (21). For convenience, we wrote the solution of (20) parametrically, which enabled us to find f(x). One form of the function f(x) was given by

(27) $$f\left(x\right)∼\frac{1}{x^{5}}{\left(1-\frac{1}{x}\right)}^{-15/7},$$

in (24), so that the first integral could be written parametrically as (25). Remarkably, we have obtained a new solution of the Emden–Fowler Equation (4) with a new functional dependence of f(x) given in (27). Note that the solutions by Stephani [30], Srivastava [31], and Maharaj et al. [34] are not regained from our solution. Thus, our results complement those and, together, constitute a more complete analysis of (4). This first integral may be related to the geometrical structure of the Emden–Fowler equation. The complexity of a self-gravitating relativistic shear-free fluid has been shown to be related to a first integral arising from the integration of the Emden–Fowler equation in our treatment.

Extensions of the approach in this paper to include charged matter distributions may also lead to useful results. In the presence of charge, the Emden–Fowler Equation (4) becomes

(28) $$y_{xx}=f\left(x\right)y^{2}+g\left(x\right)y^{3},$$

where g(x) is related to the charge distribution (see Wafo Soh and Mahomed [38]). Equation (28) arises from the analysis of the Einstein–Maxwell field equations. It may be possible to consider extensions of this work to include anisotropy and dissipation, in addition to the electromagnetic field. For this physical scenario, the generalization of (28) will involve terms containing the heat flux and anisotropic pressure. The subsequent analysis of the resulting differential equation will involve an extension of the approach developed in this paper for a shear-free uncharged relativistic fluid. For a recent analysis of charged fluids with anisotropy and dissipation relevant to radiating stars, see the analysis of Abebe and Maharaj [46], where the geometric approach of Lie symmetries provided new solutions. The complexity of a self-gravitating relativistically charged, anisotropic, and dissipative fluid will then be related to a first integral arising from the integration of the generalized Emden–Fowler equation. This suggests that there may be a deeper connection between general matter fluids, first integrals, and complexity. This deserves further investigation.

## Data Availability

This manuscript has no associated data.

## Appendix A. Intergation of ${\bar{L}}_{xxx}=C_{0}x{\bar{L}}^{-5/2}$

In this appendix, we show how Equation (20) can be integrated to yield the solution (21). The nonlinear differential Equation (20) may be written as

$$\bar{L}{\bar{L}}_{xxx}=C_{0}x{\bar{L}}^{-\frac{3}{2}}.$$

The left hand side can be expressed in the exact form

$${\left(\bar{L}{\bar{L}}_{xx}\right)}_{x}-{\bar{L}}_{x}{\bar{L}}_{xx}={\left(\bar{L}{\bar{L}}_{xx}\right)}_{x}-\frac{1}{2}{\left({\bar{L}}_{x}^{2}\right)}_{x}.$$

Integrating, we obtain

$$\bar{L}{\bar{L}}_{xx}-\frac{1}{2}{\bar{L}}_{x}^{2}=C_{1}+C_{0}\int x{\bar{L}}^{-\frac{3}{2}}dx,$$

where $C_{1}$ is constant. Again, focussing on the left hand side, we can write

$$x{\bar{L}}^{-\frac{3}{2}}\left(\bar{L}{\bar{L}}_{xx}-\frac{1}{2}{\bar{L}}_{x}^{2}\right)=x{\left({\bar{L}}^{\frac{1}{2}}\right)}_{xx}={\left[x{\left({\bar{L}}^{\frac{1}{2}}\right)}_{x}\right]}_{x}-{\left({\bar{L}}^{\frac{1}{2}}\right)}_{x},$$

and so we have the equation

$${\left[x{\left({\bar{L}}^{\frac{1}{2}}\right)}_{x}\right]}_{x}-{\left({\bar{L}}^{\frac{1}{2}}\right)}_{x}=C_{1}x{\bar{L}}^{-\frac{3}{2}}+C_{0}x{\bar{L}}^{-\frac{3}{2}}\int x{\bar{L}}^{-\frac{3}{2}}dx,$$

where we have absorbed the factor of $\frac{1}{2}$ in $C_{0}$ and $C_{1}.$ This can be easily integrated to yield

(A1) $$x{\left({\bar{L}}^{\frac{1}{2}}\right)}_{x}-{\bar{L}}^{\frac{1}{2}}=C_{2}+C_{1}\int x{\bar{L}}^{-\frac{3}{2}}dx+\frac{1}{2}C_{0}{\left(\int x{\bar{L}}^{-\frac{3}{2}}dx\right)}^{2},$$

where $C_{2}$ is a new constant. The equation above is not in standard form. However, it is still possible to make progress. When multiplied by a factor $x{\bar{L}}^{-\frac{3}{2}}$, Equation (A1) above can be written as

$$\begin{array}{ccc} \frac{1}{2}x^{2}{\bar{L}}^{-2}{\bar{L}}_{x}-x{\bar{L}}^{-1} & = & C_{2}x{\bar{L}}^{-\frac{3}{2}}+C_{1}x{\bar{L}}^{-\frac{3}{2}}\int x{\bar{L}}^{-\frac{3}{2}}dx+\frac{1}{2}C_{0}x{\bar{L}}^{-\frac{3}{2}}{\left(\int x{\bar{L}}^{-\frac{3}{2}}dx\right)}^{2}. \end{array}$$

The left hand side can be written as a total derivative, and we have

$$\begin{array}{ccc} {\left(-\frac{1}{2}x^{2}{\bar{L}}^{-1}\right)}_{x} & = & C_{2}x{\bar{L}}^{-\frac{3}{2}}+C_{1}x{\bar{L}}^{-\frac{3}{2}}\int x{\bar{L}}^{-\frac{3}{2}}dx+\frac{1}{2}C_{0}x{\bar{L}}^{-\frac{3}{2}}{\left(\int x{\bar{L}}^{-\frac{3}{2}}dx\right)}^{2}. \end{array}$$

The integral of this equation is

$$\begin{array}{ccc} -\frac{1}{2}x^{2}{\bar{L}}^{-1} & = & C_{3}+C_{2}\left(\int x{\bar{L}}^{-\frac{3}{2}}dx\right)+\frac{1}{2}C_{1}{\left(\int x{\bar{L}}^{-\frac{3}{2}}dx\right)}^{2}+\frac{1}{6}C_{0}{\left(\int x{\bar{L}}^{-\frac{3}{2}}dx\right)}^{3}, \end{array}$$

where $C_{3}$ is constant. This can be simplified to

$$\begin{array}{ccc} x^{2}{\bar{L}}^{-1} & = & -2C_{3}-2C_{2}\left(\int x{\bar{L}}^{-\frac{3}{2}}dx\right)-C_{1}{\left(\int x{\bar{L}}^{-\frac{3}{2}}dx\right)}^{2}-\frac{1}{3}C_{0}{\left(\int x{\bar{L}}^{-\frac{3}{2}}dx\right)}^{3}. \end{array}$$

Redefining the constants in the above equation, we can write it as

$$\begin{array}{ccc} x^{2}{\bar{L}}^{-1} & = & {\bar{C}}_{3}+{\bar{C}}_{2}\left(\int x{\bar{L}}^{-\frac{3}{2}}dx\right)+{\bar{C}}_{1}{\left(\int x{\bar{L}}^{-\frac{3}{2}}dx\right)}^{2}+{\bar{C}}_{0}{\left(\int x{\bar{L}}^{-\frac{3}{2}}dx\right)}^{3}, \end{array}$$

where ${\bar{C}}_{3}=-2C_{3},$ ${\bar{C}}_{2}=-2C_{2},$ ${\bar{C}}_{1}=-C_{1}$ and ${\bar{C}}_{0}=-\frac{C_{0}}{3}.$ Therefore, the third order Equation (20) has been integrated to yield the solution (21).
